# Aptamers and Their Biological Applications

**DOI:** 10.3390/s120100612

**Published:** 2012-01-09

**Authors:** Kyung-Mi Song, Seonghwan Lee, Changill Ban

**Affiliations:** Department of Chemistry, Pohang University of Science and Technology, San31, Hyoja-dong, Pohang, Gyeongbuk 790-784, Korea; E-Mails: kmsong@postech.ac.kr (K.-M.S.); s.lee@postech.ac.kr (S.L.)

**Keywords:** aptamer, SELEX, *in vitro* selection, aptasensor, diagnosis, biosensor

## Abstract

Recently, aptamers have attracted the attention of many scientists, because they not only have all of the advantages of antibodies, but also have unique merits, such as thermal stability, low cost, and unlimited applications. In this review, we present the reasons why aptamers are known as alternatives to antibodies. Furthermore, several types of *in vitro* selection processes, including nitrocellulose membrane filtration, affinity chromatography, magnetic bead, and capillary electrophoresis-based selection methods, are explained in detail. We also introduce various applications of aptamers for the diagnosis of diseases and detection of small molecules. Numerous analytical techniques, such as electrochemical, colorimetric, optical, and mass-sensitive methods, can be utilized to detect targets, due to convenient modifications and the stability of aptamers. Finally, several medical and analytical applications of aptamers are presented. In summary, aptamers are promising materials for diverse areas, not just as alternatives to antibodies, but as the core components of medical and analytical equipment.

## 1. Introduction

Aptamers are oligonucleotides, such as ribonucleic acid (RNA) and single-strand deoxyribonucleic acid (ssDNA) or peptide molecules that can bind to their targets with high affinity and specificity due to their specific three-dimensional structures. Especially, RNA and ssDNA aptamers can differ from each other in sequence and folding pattern, although they bind to the same target. The concept of joining nucleic acids with proteins began to emerge in the 1980s from research on human immunodeficiency virus (HIV) and adenovirus. It indicated that these viruses encode a number of small structured RNAs that bind to viral or cellular proteins with high affinity and specificity [1]. In the case of HIV, a short RNA ligand called the trans-activation response (TAR) element promotes trans-activation and virus replication by binding with the viral Tat protein [2]. The adenovirus also has a short RNA aptamer, virus-associated (VA)-RNA, that regulates translation [3,4]. Substantive studies on aptamers have progressed since the *in vitro* selection process called Systematic Evolution of Ligands by EXponential enrichment (SELEX) was first reported by both Gold’s group and Szostak’s group in 1990 [5,6]. Due to the development of SELEX, which is now a basic technique for the isolation of aptamers, many aptamers could be directly selected *in vitro* against various targets, from small biomolecules to proteins and even cells [7].

**Figure 1.**
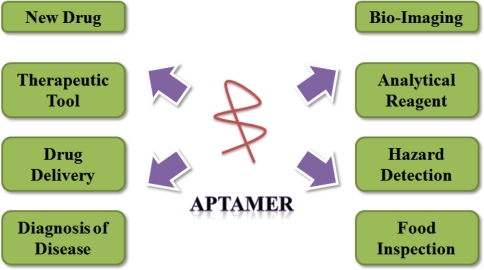
Various application fields of aptamers.

Aptamers have been studied as a bio-material in numerous investigations concerning their use as a diagnostic and therapeutic tool and biosensing probe, and in the development of new drugs, drug delivery systems, *etc*. (Figure 1). There have been attempts to search for aptamers that are specific to targets involved in various diseases, such as cancer and viral infection. Developed aptamers have been studied primarily for applications as diagnostic or therapeutic tools. In 2004, the approval by the Food and Drug Administration (FDA) of Macugen, a vascular endothelial growth factor (VEGF)-specific aptamer, for the treatment of neovascular (wet) age-related macular degeneration (AMD), is a prominent landmark in the application of aptamers [8]. Since then, aptamer technology has been regarded as more effective and more authoritative, and the number of studies on the applications of aptamers is rapidly increasing. There are many fields to which aptamers may be applied, as outlined below.

The use of antibodies as the most popular class of molecules for molecular recognition in a wide range of applications has been around for more than three decades. Aptamers are widely known as a substitute for antibodies, because these molecules overcome the weaknesses of antibodies. The advantages of aptamers compared with antibodies are described in the following sections [7]:

1. High stability of aptamers: It is well known that proteins are easily denatured and lose their tertiary structure at high temperatures, while oligonucleotides are more thermally stable and maintain their structures over repeated cycles of denaturation/renaturation. Hence, the greatest advantage of oligonucleotide-based aptamers over protein-based antibodies is their stability at elevated temperatures. Aptamers recover their native conformation and can bind to targets after re-annealing, whereas antibodies easily undergo irreversible denaturation [9]. Thus, aptamers can be used under a wide range of assay conditions.
2. Production of aptamers (synthesis/modification): The identification and production of monoclonal antibodies are laborious and very expensive processes involving screening of a large number of colonies. Additionally, the clinical commercial success of antibodies has led to the need for very large-scale production in mammalian cell culture [10]. Moreover, immunoassays are required to confirm the activity of the antibodies in each new batch, because the performance of the same antibody tends to differ depending on the batch. However, aptamers, once selected, can be synthesized in quantity with great accuracy and reproducibility via chemical reactions. These chemical processes are more cost effective than the production of antibodies. Furthermore, aptamers can be easily modified by various chemical reactions to increase their stability and nuclease resistance [11,12]. Additionally, it is possible to introduce signal moieties, such as fluorophores and quenchers, that greatly facilitate the fabrication of biosensors.
3. Low immunogenicity of aptamers: Aptamers usually seem to be low-immunogenic and low-toxic molecules, because nucleic acids are not typically recognized by the human immune system as foreign agents. However, antibodies are significantly immunogenic, which precludes repeat dosing [13]. The Eyetech Study Group demonstrated that a VEGF-specific aptamer displayed little immunogenicity when given to monkeys in 1,000-fold higher doses [14,15].
4. Variety of target: In instances of toxins or molecules that do not elicit strong immune responses, it is difficult to identify and produce antibodies, but aptamers can be generated in sufficient numbers. Moreover, aptamers show a high affinity and specificity for some ligands that cannot be recognized by antibodies, such as ions or small molecules, indicating that employing aptamers as the recognition components may markedly broaden the applications of the corresponding biosensors [12].

Based on the many advantages described above, aptamers are considered to be an alternative to antibodies in many biological applications.

## 2. *In Vitro* Selection

### 2.1. General

**Figure 2.**
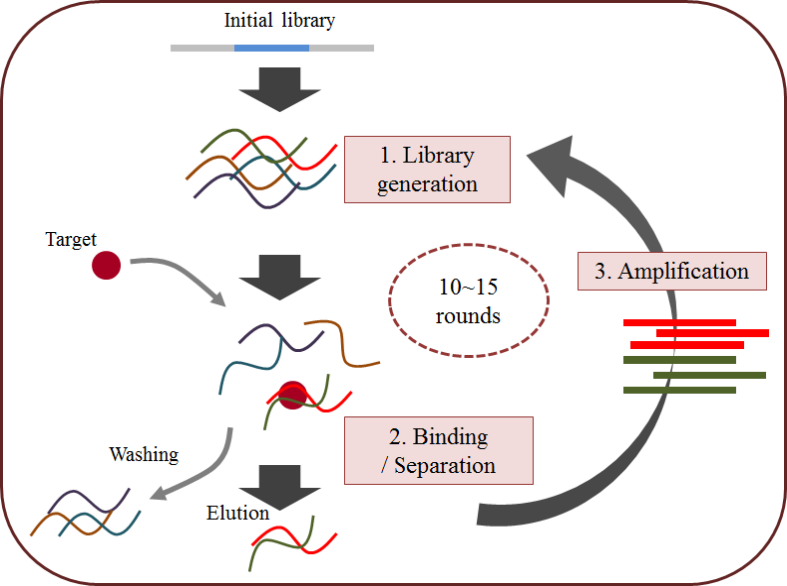
The general SELEX strategy. Starting with combinatorial libraries (first step), the specific binders are isolated by an iterative process of ligand binding, elution (second step), and amplification (third step).

As mentioned above, SELEX or *in vitro* selection is a technique used to isolate aptamers with high affinity for a given target from approximately 10^12^–10^15^ combinatorial oligonucleotide libraries. In general, the SELEX process is comprised of three steps that are repeated in order to search for nucleotides that are better able to bind to the target (Figure 2) [16]. In the first step (library generation), a library, except the initial compound in the library, is converted into single-strand nucleotides that consist of random sequence regions, usually 30–40 mers, flanked by the primer binding site. In the second step (binding and separation), the target-bound library components are separated from the unbound components. This step is generally coupled with several other methods to make selection of the target or the library easy and rapid. Finally, in the third step (amplification), the target-bound library component is amplified by the PCR to create a new library to be used in the next round. Aptamers are continuously developed through this on-going process, and their characteristics are identified using various biological assays.

### 2.2. Nitrocellulose Membrane Filtration-Based SELEX

A nitrocellulose membrane is often used to immobilize proteins in Western blots and atomic force microscopy (AFM) because it provides simple and rapid protein immobilization by its non-specific affinity for amino acids. In 1968, a method using nitrocellulose membranes was employed by Kramlova’s group to easily and quickly separate a protein from RNA molecules [17]. This method has been developed as a research tool for separating proteins from many other components and for immobilizing proteins, which can then react with other biomolecules. When the SELEX method was initially established by Gold’s group, the aptamer against the T4 DNA polymerase was obtained using a strategy that was based on the use of a nitrocellulose membrane [6]. Because the targets were primarily proteins in the early stage of SELEX, the use of a nitrocellulose membrane was applied during the separation step. However, these membranes have some limitations, such as being incapable of binding small molecules and peptides, and they generally require at least 12 selection rounds [18,19].

### 2.3. Affinity Chromatography and Magnetic Bead-Based SELEX

**Figure 3.**
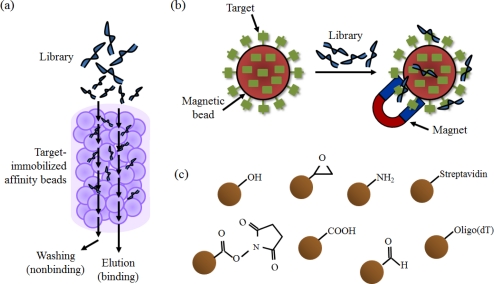
(**a**) A schematic illustration of the selection step from a library using an affinity column; (**b**) The process of the selection step using magnetic beads; (**c**) Several types of functional group-activated beads such as tosyl-activated beads and epoxy-activated beads.

Affinity chromatography is a method for separating the components from a biochemical mixture. It is primarily used for the purification of recombinant proteins, based on a highly specific biological interaction, such as that between a receptor and a ligand, or an antigen and an antibody. The immobile phase is generally composed of agarose-based beads, and the beads are packed onto a column for the washing and elution processes. In the binding and separation steps of SELEX, affinity chromatography assists in the selection of only the library components with an affinity for the target, by immobilizing target molecules on the beads (Figure 3(a)). In the case of immobilization of proteins, various tags such as glutathione S-transferase (GST) and the His-tag are utilized and, in the case of small organic molecules, the targets are covalently fixed on the beads via a chemical reaction, such as coupling with EDC [20]. Thus, SELEX for small molecules, as well as proteins, can be carried out using this method. Several aptamers were selected using this method by making an affinity column containing target-immobilized beads [20–22]. However, the disadvantage of this method is that it cannot be applied if the target lacks the affinity tag or the functional group needed for coupling to the beads.

Magnetic beads are also used to immobilize the target via an interaction or a chemical reaction between an affinity tag and the substrate on the beads. The use of magnetic beads is an especially powerful tool for easy and rapid isolation of target-immobilized beads with a magnet (Figure 3(b,c)). Recently, aptamer selection has been involved in linking this magnetic bead-based strategy to the SELEX technique. Particularly because the library bound to the target is easily and simply separated from the unbound one by a magnet, many attempts at selecting target specific aptamer have followed [23–25].

### 2.4. Capillary Electrophoresis-Based SELEX

**Figure 4.**
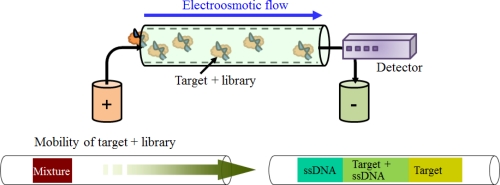
Fundamental principle of the selection step using CE. Aptamers are selected based on the difference in mobility due to charge and mass.

Capillary electrophoresis (CE) possesses several appealing advantages over the other analytical separation methods in the aspects of speed, resolution, capacity, and minimal sample dilution. This method can separate ionic species by their charge, frictional forces, and hydrodynamic radius under the influence of an electric field [18]. With this method, an aptamer can be selected by a mobility shift among the mixture of a target, the library, and the target-library complex (Figure 4). In particular, the greatest virtue of applying this method to SELEX is that the successful selection of the aptamer can be achieved within very few rounds, generally 2–4 rounds, compared to other methods. In Bowser’s group, aptamers for neuropeptide Y and human IgE were obtained after only four rounds of selection [26,27].

### 2.5. Microfluidic-Based SELEX

In order to select an aptamer more effectively, SELEX using a microfluidic or chip system was developed [28]. Since this method is mainly processed on a chip, it is able to enhance selection efficiency on a small scale. For example, the DNA aptamer-specific bound to neurotoxin type B was obtained after a single round of selection using the Continuous-flow Magnetic Activated Chip-based Separation (CMACS) device designed by Soh’s group [29]. Also, the screening of C-reactive protein (CRP) specific aptamers was performed automatically utilizing a microfluidic system and magnetic beads conjugated with CRP [30]. Microfluidic technology-based SELEX is catching on as an advanced method to select aptamers rapidly and automatically.

### 2.6. Cell-SELEX

Cell-SELEX is aimed at searching for an aptamer against a whole cell, whereas the primary targets of other SELEX methods are single highly-purified proteins. In other words, the targets of Cell-SELEX are extracellular proteins on the cell surface or unique structures of the cell. In the majority of cases, Cell-SELEX processes have washing (for adhesive cells) or centrifugation (for suspension cells) steps during the separation of aptamers, because target immobilization is not practicable in the solid phase. In addition, counter selections are necessary in each round to avoid selection of aptamers that non-specifically recognize the cell surface and that are commonly located at the surfaces of many cells. Therefore, this method is complicated, because of the impossibility of immobilizing targets, and due to counter selection. However, the resulting aptamers, once selected, are powerful for cell-specific diagnosis, cell-targeted drug delivery, and cell-specific therapy.

Recently, in Kobatake’s group, a DNA aptamer for the SBC3, an adherent small cell lung cancer (SCLC) cell line, was identified using Cell-SELEX. The aptamer could be a potential SBC3-specific marker because this cell line does not express the common biomarker pro-GRP that is used to diagnose SCLC [31]. In addition, Tan’s group has selected a series of aptamers that bind to two types of ovarian cancer cells: ovarian clear cell adenocarcinomas (TOV-21G) and ovarian serous adenocarcinomas (CAOV-3) [32]. In 2003, Gold’s group isolated a GBI-10 aptamer for the U251 cell line, which is derived from a human glioblastoma, by performing Cell-SELEX. It was determined that a binding partner interacting with the GBI-10 aptamer is the tenascin-C protein on the cell surface [33].

### 2.7. Other Method-Based SELEX

Several methods, such as AFM, electrophoretic mobility shift assays (EMSA), and surface plasmon resonance (SPR), have been performed in connection with SELEX [34–37]. Although these strategies have the advantage of reducing the number of selection rounds, the effectiveness of these methods in selecting the aptamer has not been clearly demonstrated.

## 3. Aptamer-Based Sensor

### 3.1. General

A biosensor that is based on aptamers as a recognition element is called an aptasensor [38]. These aptasensors can be constructed through a variety of methodologies, including electrochemical biosensors, optical biosensors, and mass-sensitive biosensors.

### 3.2. Electrochemical Aptasensor

An electrochemical analysis is an attractive platform, because it offers high sensitivity, compatibility with novel microfabrication technologies, inherent miniaturization, and low cost. Therefore, various electrochemical aptasensors have been fabricated using several techniques, including EIS, potentiometry with ISEs, ECL, CV, and DPV [39–44].

**Figure 5.**
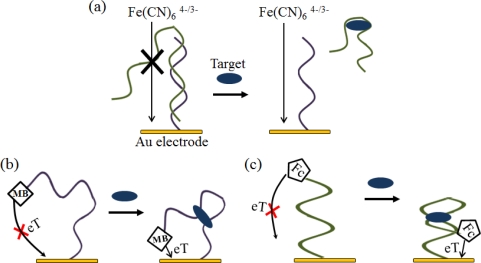
Examples of electrochemical aptasensors. (**a**) A schematic representation of the electrochemical aptasensor using Fe(CN)_6_^4−/3−^. Part of the aptamer was a hybridized aptamer with complementary DNA, which was immobilized on the gold surface. In the presence of the target, the aptamer was followed by binding with the target, to decrease the amount of the aptamer on the electrode surface; (**b**) A schematic representation of the electrochemical aptasensor using MB. In the presence of the target, the aptamer folds into the target-binding three-way junction, altering the electron transfer (eT) and increasing the observed reduction peak; (**c**) A schematic representation of the electrochemical aptasensor using Fc. In the presence of the target, the aptamer folds into the restricted hairpin structure, and this conformational change results in increased efficiency of eT between the Fc probe and the electrode surface.

To enhance the sensitivity, an AuNRs- or AuNPs-modified conducting polymer was used as a material for immobilizing on the electrode [45,46]. Additionally, electroactive reporters, such as methylene blue (MB), ferrocence, ferrocence-bearing polymers, ruthenium complexes, and Fe(CN)_6_^4−/3−^, are used for signal transduction (Figure 5) [41,47–50]. In Gothelf’s group, a signal-on electrochemical aptasensor was designed for theophylline detection using a redox-active Fc moiety, which could transfer electrons with the electrode surface by DPV or CV [51].

### 3.3. Fluorescence-Based Optical Aptasensor

**Figure 6.**
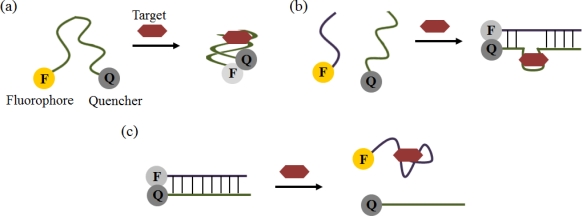
Schematic illustrations of optical aptasensors using fluorescence. (**a**) The simplest format of a quenching aptamer beacon. The binding of the target stabilizes the stem and brings the quencher and fluorophore in close proximity, resulting in fluorescence decrease; (**b**) Assembly aptamer beacon. The binding of the target brings the oligomers together and leads to ternary complex stabilization; (**c**) Disassembly aptamer beacon. The target binding induces an antisense displacement and results in a fluorescence increase.

Aptamers have been used as bio-probes in optical sensors based primarily on the incorporation of a fluorophore or a nanoparticle. In the case of fluorescence detection, the simplest format is to label the aptamers with both a quencher and a fluorophore. A cocaine-specific aptamer-based strategy was able to detect the target using a FRET signal between fluorescein and DABCYL moieties (Figure 6(a)) [52]. Aptamer beacons are probes that can monitor the presence of the target in solutions using fluorophore-quencher pairs. They can change into two or more conformations, such as hairpin shape or a hybridization form, by target binding. More complicated formats utilizing quaternary structural rearrangements that result in the assembly or disassembly of aptamers were also developed for fluorescence signaling (Figure 6(b,c)) [53–55]. Additionally, many nano-materials, including QDs, AuNPs, CNTs, graphene oxide (GO), polymer nanobelts, and coordination polymers, have been investigated for their fluorescence-quenching effect instead of using a more traditionally quencher [56–62].

### 3.4. Colorimetric-Based Optical Aptasensor

**Figure 7.**
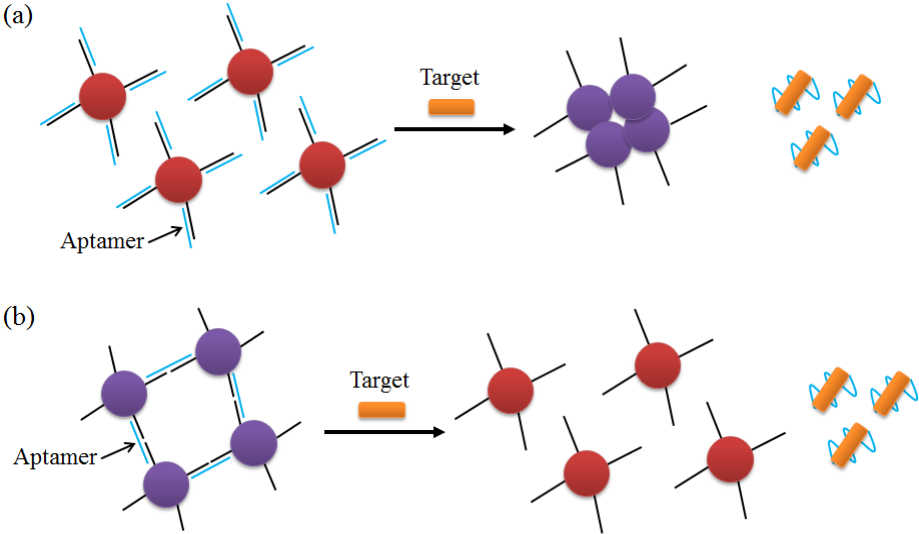
Schematic illustrations of optical aptasensors using AuNPs. (**a**) Aptamer release and AuNP aggregation by target binding; (**b**) Aptamer release and AuNP disaggregation by target binding.

AuNPs or several polymers that cause color changes, can be applied as novel reagents for the optical detection technique called colorimetry. The highly negatively-charged ssDNA (complementary strand of the aptamer), which is separated from the aptamer by interaction between the aptamer and the target, is stabilized against aggregation, and a color change occurs in conjunction with this phenomenon (Figure 7(a)) [63]. In contrast to this method, the AuNP disaggregation method was employed to detect ATP, cocaine, Pb^2+^, and K^+^ (Figure 7(b)) [64,65]. After hybridizing under the two kinds of oligonucleotides, the aptamer and a linker, the cross-linked AuNPs are released, because the aptamer undergoes a conformational change in the presence of the target. These colorimetric strategies have the virtue of being easily visible to the naked eye without the need for complicated instruments.

### 3.5. Other Aptasensors

**Figure 8.**
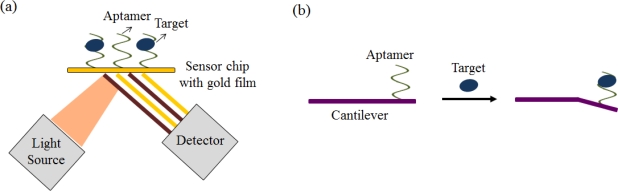
(**a**) SPR-based aptasensor and (**b**) microchannel cantilever-based aptasensor.

Numerous other aptasensors have been exploited in combination with various types of analytical equipment, such as those used for SPR, surface acoustic wave (SAW), QCM, and microchannel cantilever sensors (Figure 8) [66–72]. A nanoaptasensor using a single wall nanotube (SWNTs) device was developed for detecting small molecules [73]. These methods generally do not require target labeling; they only observe the differential changes in optical properties and mass. Therefore, these methods are primarily appropriate for relatively large molecules but not for small molecules, including organic molecules, toxins, and metabolites [74,75].

**Figure 9.**
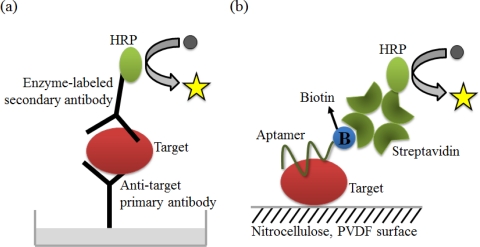
Schematic illustrations of (**a**) the ELISA method; and (**b**) the ALISA method.

ELISA, one of the major clinical diagnostic tests available, is a versatile technique to detect almost any protein or peptide with high sensitivity. One version of ELISA, commonly referred to as sandwich ELISA, involves the simultaneous use of two antibodies or analyte-binding receptor proteins to capture the analyte or target and to report the target detection (Figure 9(a)). An aptamer-linked immobilized sorbent assay (ALISA) was introduced by Kiel’s group. They demonstrated the feasibility of this method via a comparative study with ELISA using an antibody (Figure 9(b)). It is important to note that aptamers have an unlimited potential to circumvent the limitations associated with antibodies [76].

**Figure 10.**
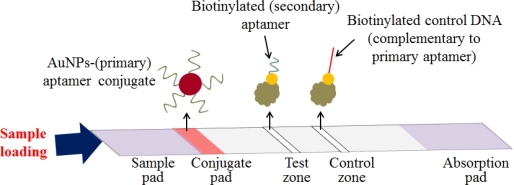
A schematic illustration of an AuNPs-based strip assay.

In addition to ELISA, another common tool for clinical diagnosis is RDT, which is a rapid and simple method for point-of-care testing (POCT). RDTs for the diagnosis of infectious diseases, such as malaria and influenza, are already in use as commercially available tests. Because of the high sensitivity of the aptamer, several aptamer-based RDT methods, for many biomarkers related to diseases, have been introduced for use in early diagnosis or convenient POCT. In particular, Liu’s group has developed a dry-reagent strip biosensor based on aptamers and functionalized AuNPs for use in thrombin analysis (Figure 10). In this study, the sensor is subject to visual detection of protein within minutes, with sensitivity and specificity that are superior to those of the antibody-based strip sensor [77]. The target interacts with the AuNP-primary aptamer conjugate, while the sample solution containing the target migrates onto each pad. Then, the target-aptamer-AuNP complexes are captured by the secondary aptamer that is immobilized in the test zone. Finally, a red band can be observed on the test zone due to the accumulation of AuNPs.

### 4.2. Therapy (New Drugs)

As mentioned above, Macugen, developed by Pfizer and Eyetech, is already commercially available for AMD. This drug is a pegylated aptamer, a single strand of nucleic acid with specificity to VEGF165, which plays a critical role in angiogenesis and permeability [78]. Additionally, Regado Bioscience has developed REG1 as a new aptamer drug for anticoagulation, and this aptamer drug is currently in Phase II clinical trials. REG1 consists of two components: RB006 (coagulation factor IXa-specific aptamer) and RB007 (oligonucleotide antidote of the RB006 aptamer) [79]. RB006 is a 2′-ribo purine/2′-fluoro pyridimidine aptamer and is conjugated to a 40 kDa PEG to protect the aptamer against nuclease-mediated degradation. In addition, many aptamer-based drugs, such as AS1411 (a nuclein-specific aptamer) for acute myeloid leukemia, ARC1779 (a vonWillebrand factor-specific aptamer) for carotid artery disease, and NU172 (a thrombin-specific aptamer) for anticoagulation, are currently in a clinical trials [80–86].

### 4.3. Drug Delivery System

**Figure 11.**
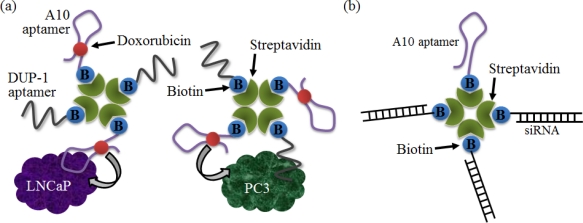
(**a**) A schematic illustration of the active targeting of the drug doxorubicin to prostate cancer cells using the dual-aptamer (A10 and DUP-1) complex. Doxorubicin that is bound to an A10 aptamer can enter both PSMA(+) and PSMA(−) prostate cancer cells via the dual-aptamer complex; (**b**) Design of an siRNA-aptamer conjugate via a modular streptavidin bridge using an anti-PSMA aptamer for prostate cancer cells (LNCaP).

Aptamers that bind to internalized cell surface receptors have been exploited to deliver drugs and a variety of other cargo into cells. For example, the prostate-specific membrane antigen (PSMA) is an important prostate cancer marker [87]. The dual aptamer probe—an A10 aptamer for PSMA(+) prostate cancer cells, and a DUP-1 aptamer for PSMA(−) prostate cancer cells—was invented, and a drug-loaded dual aptamer complex was constructed by loading doxorubicin, an anticancer drug, onto the A10 aptamer strand (Figure 11(a)). As a result, the doxorubicin can be effectively introduced into the prostate cancer cells [88].

Small interfering RNAs (siRNAs) have received considerable attention recently as a new class of therapeutics for a variety of diseases. The role of siRNA is to induce an RNA interference (RNAi) pathway, which regulates the expression of a specific gene. In particular, it is important to efficiently and safely deliver siRNAs into specific cells in order for the therapeutic use of the siRNAs to be effective. Levy’s group introduced an siRNA-aptamer conjugate via a modular streptavidin bridge using an anti-PSMA aptamer for prostate cancer cells (LNCaP) (Figure 11(b)) [89]. As a result, the conjugates could be added into the LNCaP cells within 30 min, and the siRNA-mediated inhibition of gene expression was observed to be effective.

### 4.4. Bio-Imaging

Another application is bio-imaging, using an aptamer that is conjugated to a fluorophore, a QD, or other materials such as gadolinium, which is useful for magnetic resonance imaging (MRI). Using aptamers as imaging agents has the advantage of their being non-toxic, because oligonucleotide moieties are present in the human body. Additionally, as aptamers have high specificity for their target, accurate targeting, and rapid diffusion through the blood circulation, use of these molecules can increase the certainty of the results obtained during diagnosis or clinical analysis. Based on these advantages, aptamers have been studied as imaging agents for cell imaging as well as single-protein imaging.

Kim’s group published the results of C6 cell imaging using a Cy3-labeled AS1411 aptamer, which included a chemical modification of 5-(*N*-benzylcarboxyamide)-2′-deoxyuridine (called 5′-BzdU) on a thymidine base [90]. The AS1411 aptamer is specific for the nucleolin transmembrane protein in cancer cells. Specifically, this group improved the aptamer’s binding affinity to its target via a chemical modification. The cell imaging of the modified Cy3-labeled AS1411 aptamer was more efficient for the C3 cells than the original Cy3-labeled AS1411 aptamer.

The QD-A10 and DUP-1 aptamer complex, which is specific for PSMA(+) and PSMA(−) prostate cancer cells (LNCaP and PC3), was shown by imaging to bind only to prostate cancer cells and not to normal (PNT2) or other cancer cells [91]. Additionally, an aptamer specific for p68 in liver tumors and an aptamer specific for small cell lung cancer (SCLC) cells were also demonstrated to have potential as bio-imaging probes [92–94].

### 4.5. Western Blot Analysis

**Figure 12.**
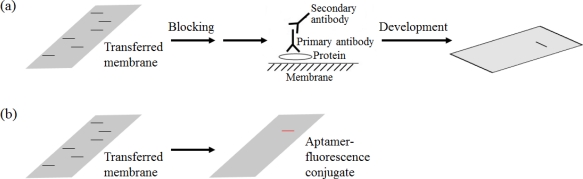
(**a**) The conventional Western blot analysis, (**b**) The aptamer-based Western blot analysis.

A Western blot analysis is an analytical technique routinely used to quantify specific proteins (Figure 12(a)). The procedure includes complicated and elaborate steps and requires many reagents, such as two types of antibodies. There are now many reagent companies that specialize in providing antibodies against tens of thousands of different proteins. Hah’s group published a new aptamer-based Western blot strategy that has reduced the procedure to one step, and easily detects the target protein using only one aptamer (Figure 12(b)). Instead of two types of antibodies, the QD-conjugated RNA aptamer specific for the His-tag was employed. This method has the advantages of requiring less time, not requiring antibodies or ^32^P, and introducing the possibility of multiplexing detection [95].

### 4.6. Aptamer Affinity Chromatography

Immunoaffinity purification is a general laboratory technique used in many scientific fields, that relies on the interaction between an antigen and an antibody to purify target proteins. The use of an aptamer in chromatography has many advantages over the use of an antibody, including an equal or superior affinity and specificity to the target, a smaller size, easier modification and immobilization, better stability, and higher reproducibility. Drolet’s group developed an aptamer affinity chromatography system for human l-selectin. The recombinant human l-selectin-Ig fusion protein was successfully purified from Chinese hamster ovary (CHO) cell-conditioned medium using this aptamer affinity column [96]. Additionally, in Le’s group, it was demonstrated that a designed sandwich aptamer affinity chromatography using two aptamers improved the sensitivity and selectivity for thrombin [97].

## 5. Conclusions

In this review article, the advantages of aptamers and their applications have been presented through descriptions of several excellent investigation projects. An aptamer is a convincing substitute for an antibody because it is very stable under hot or hazardous conditions. Since aptamers are mostly oligonucleic acids, they can easily be synthesized in quantity with high purity, and can be modified with various molecules using simple chemical reactions. Therefore, aptamers have shown great strength in a wide range of applications. Moreover, aptamers are non-immunogenic and non-toxic materials that can be exploited in medical applications, such as diagnosis and treatment of diseases. It is also noteworthy that the types of target molecules are unlimited. Diverse SELEX methods have been designed as the targets to select aptamers conveniently and quickly. Affinity chromatography and magnetic bead-based SELEX are powerful techniques for relatively large molecules such as proteins, because of their high affinity to the target and easy separation from dispensable molecules. Besides, capillary electrophoresis-based SELEX is suitable for small molecules and is a rapid method. Aptamers against biomarker related diseases can be used to diagnose these diseases. In the ALISA and RDT methods, aptamers have been used as capture-probe molecules, based on their affinity and selectivity to the targets. As a result, sensors have shown excellent performance in diagnosis of diseases. For the detection of small molecules, aptamers will be utilized pivotally, because antibody-based detection has considerable limitations, while the applications of aptamers are relatively broad. Numerous sensor techniques incorporating aptamers have been described, such as electrochemical, colorimetric, optical, and mass-sensitive methods. These aptasensors are being employed in various fields, including the medical and industrial fields. Beyond this, the use of aptamers as drugs, therapeutics, bio-imaging materials, and analytical reagents have been investigated and, as a result, the drug Macugen came into the world. In conclusion, it is certain that aptamers are a strong and versatile alternative to antibodies. Of course, aptamers still have issues that need to be resolved for purposes of biological applications. For example, if they are used as agents for *in vivo* applications, unmodified aptamers are highly susceptible to degradation by several nucleases, and their small size makes them liable to renal filtration, but it can be expected that the potential of aptamers for bio-applications will increase, based on their merits. Thus, further investigations into aptamers and aptasensor systems need to be carried out in order to quckly overcome existing limits.

## Appendix

**Table A1.** Aptamer-based sensor.

	**Method**	**Material**	**Reference**
Electrochemical aptasensor	Electrochemical impedance spectroscopy (EIS)	Label-free	[39]
		Fe(CN)_6_^4−/3−^	[45]
		Ferrocene	[46]

	Potentiometry-based ion-selective electrodes (ISEs)	CdS quantum dot (QD)	[40]

	Electrogenerated chemiluminescence (ECL)	Ru(bpy)_3_^2+^	[41]

	Cyclic voltammetry (CV)	MB	[42]
		Pyrroquinoline quinone (PQQ)	[43]
		Ferrocene	[49]
		Gold nanorod (AuNRs)-modified conducting polymer	[47]

	Differential pulse voltammetry (DPV)	Ferrocene	[51]
		AuNRs-modified conducting polymer	[48]

Optical aptasensor	Fluorescence quenching/fluorescence resonance energy transfer (FRET)	Fluorophore/dabcyl moieties	[52]
		Fluorophore/gold nanoparticle (AuNP)	[56,58]
		Fluorophore/carbon nanotube (CNT)	[57]
		Fluorophore/graphene	[59,62]
		Fluorophore/polymer	[60]
		QD/CNT	[61]

	AuNP-based colorimetry	AuNPs	[63,64]

Other aptasensor	Quartz crystal microbalance (QCM)-based		[67,70]

	Microcantilever-based		[71,72]

	SPR-based		[74]

**Table A2.** Aptamer-based new drugs.

**New drug**	**Type**	**Target-specific aptamer**	**Reference**
Macugen	Treatment for AMD	VEGF-specific aptamer	[78]
REG1	Anti-blood clotting	Coagulation factor lxa-specific aptamer	[79]
AS1411	Anti-cancer agent	Nucleolin-specific aptamer	[80–82]
APG7909	Anti-cancer agent	TLR 9-specific aptamer	[83,84]
IMO2055			
